# Gateway-compatible tissue-specific vectors for plant transformation

**DOI:** 10.1186/s13104-015-1010-6

**Published:** 2015-03-03

**Authors:** Marta Michniewicz, Elizabeth M Frick, Lucia C Strader

**Affiliations:** Department of Biology, Washington University, St. Louis, MO 63130 USA

**Keywords:** Cloning vectors, Gateway technology, Tissue-specific

## Abstract

**Background:**

Understanding regulation of developmental events has increasingly required the use of tissue-specific expression of diverse genes affecting plant growth and environmental responses.

**Findings:**

To allow for cloning of presumptive promoters with tissue-specific activities, we created two plant expression vectors with multiple cloning sites upstream of a Gateway cassette for expression of either untagged or YFP-tagged genes of interest. For fast and easy tissue-specific expression of desired genes, we further developed an initial set of Gateway-compatible tissue-specific gene expression vectors that allow for the expression of YFP-tagged or untagged proteins driven by the *ALCOHOL DEHYDROGENASE1*, *CHLOROPHYLL A/B BINDING PROTEIN 1*, *COBRA LIKE1*, *EXPANSIN7*, *LATERAL ORGAN BOUNDARIES-DOMAIN 16*, *SCARECROW*, *UBIQUITIN10*, and *WOODEN LEG* upstream regulatory regions.

**Conclusions:**

These vectors provide an invaluable resource to the plant community, allowing for rapid generation of a variety of tissue-specific expression constructs.

## Background

Multiple genes contribute to plant development, and these contributions can vary by tissue type. Increasingly, researchers are turning to tissue-specific gene expression to gain spatial resolution of these plant processes. For example, cell-type specific gene expression has allowed for dissection of the control of many processes, including ovule development [1], cell-autonomous and non-cell-autonomous controls of photoperiodic flowering [2], and the spatial specificity of phytochrome responses [3].

The currently available systems for tissue- and cell-type-specific gene expression include either cloning desired promoters individually or using two-component systems [4-9]. In some of these two-component systems consisting of a transcription factor and a target promoter, treatment with inducers such as 17-β-estradiol [6] or ethanol [4,10,11] promotes the transcription factor binding to and activating the target promoter to allow for temporal control of gene activation in addition to the spatial control afforded by tissue-specific promoters. Although these systems have been instrumental in understanding spatial and temporal gene functions, they have the disadvantage of being unwieldy when studying tissue-specific gene rescue in higher order mutants. Additionally, the alternative of cloning individual promoters can be time-consuming.

To overcome the limitations of the current systems for creating plants with tissue-specific expression of desired genes, we have developed a set of Gateway-compatible destination vectors for tissue-specific expression to facilitate spatial analysis of gene function. This novel vector set will allow for rapid, uncomplicated construct creation for spatial examination of desired genes in higher-order mutant backgrounds.

## Findings

We have generated a vector set to provide a rapid method for creating transgenic plants expressing genes of interest in a tissue-specific manner. Vector details, including the complete DNA sequences, can be found on the website http://pages.wustl.edu/strader/vectors, which will be updated as additional vectors are created.

### Promoterless Gateway-compatible destination vectors with multiple cloning sites for creation of plant expression constructs

We generated promoterless Gateway-compatible plant expression vectors from pEarleyGate100 [12] and pEarleyGate104 [12] by replacing the cauliflower mosaic virus *35S* promoter [13] with a multiple cloning site. pEarleyGate100 [12] and pEarleyGate104 [12] were built from pFGC5941 vector (http://chromDB.org) derived from the pCAMBIA (http://www.cambia.org) vector.

**Figure 1:**
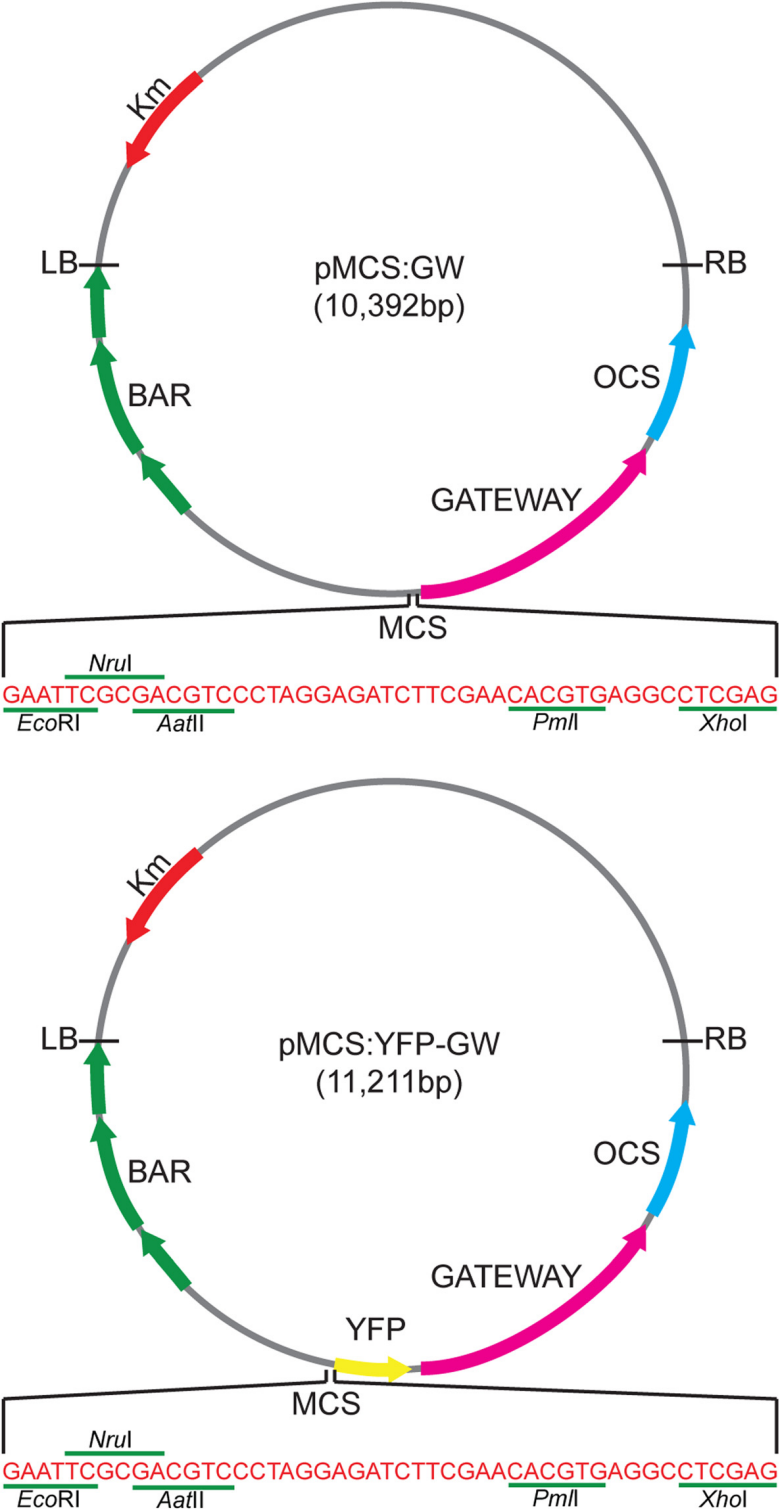
**pMCS:YFP-GW and pMCS:GW plant transformation vectors.** pMCS:YFP-GW and pMCS:GW were derived from the pEarleyGate104 [12] and pEarleyGate100 [12] vectors, respectively. The pMCS:YFP-GW and pMCS:GW vectors are binary vectors for plant transformation and confer kanamycin (Km, red arrow) and chloramphenicol (in Gateway cassette) resistance in *Escherichia coli* and kanamycin resistance (Km, red arrow) in *Agrobacterium tumefaciens*. Plants transformed with these vectors will display resistance to phosphinothricin (Basta; BAR consisting of the basta resistance gene driven by the mannopine synthase promoter and flanked by the mannopine synthase 3′ end; three green arrows). The multiple cloning site (MCS) allows for cloning of desired promoters for expression of downstream genes transferred into the vector using Gateway technology. The Gateway cassette (*att*R1, chloramphenicol resistance gene, *ccd*B, *att*R2; pink arrow) is followed by the terminator sequence from the octopine synthase gene (OCS, blue arrow). In addition, pMCS:YFP-GW has the yellow fluorescence protein (YFP, yellow arrow) gene downstram of the MCS and in-frame with the Gateway cassette. The left border (LB) and right border (RB) of the T-DNA are marked.

We created the pMCS:GW and pMCS:YFP-GW vectors from pEarleyGate100 and pEarleyGate104 by replacing the *35S* promoter regions of these starting vectors with a multiple cloning site (MCS). Because the *Eco*RI and *Xho*I sites flanking the 35S promoter are not unique in pEarleyGate100 and pEarleyGate104, we used site-directed mutagenesis to first remove the *Eco*RI site from the chloramphenicol resistance gene in the Gateway cassette of pEarleyGate100 and pEarleyGate104 and to remove the *Xho*I and *Eco*RI sites from the YFP gene of pEarlyGate104, while retaining the correct translation for the chloramphenicol resistance gene and YFP. We then excised the *35S* promoter from the mutated versions of pEarleyGate100 and pEarleyGate104 using *Eco*RI and *Xho*I and replaced it with a MCS providing *Eco*RI, *Nru*I, *Aat*II, *Pml*I, and *Xho*I sites for cloning promoters of interest. We named these vectors pMCS:GW and pMCS:YFP-GW (Figure 1).

The pMCS:GW vector consists of a T-DNA left border, followed by the Basta herbicide resistance gene, a multiple cloning site, the Gateway cassette (*att*R1, chloramphenicol resistance gene, *ccd*B, *att*R2), the 3′ sequence of the octopine synthase gene, and T-DNA right border (Figure 1). The pMCS:YFP-GW vector consists of a T-DNA left border, followed by the Basta herbicide resistance gene, a multiple cloning site, the in-frame coding region for yellow fluorescent protein (YFP), the Gateway cassette (*att*R1, chloramphenicol resistance gene, *ccd*B, *att*R2), the 3′ sequence of the octopine synthase gene, and T-DNA right border for creation of N-terminal YFP fusion proteins (Figure 1). pMCS:GW, pMCS:YFP-GW, and variants thereof confer kanamycin resistance to *Escherichia coli* and *Agrobacterium tumefaciens*. The T-DNA from these vectors confers phosphinothricin (Basta) resistance to plants.

### Gateway-compatible destination vectors for tissue-specific expression

After creating pMCS:GW and pMCS:YFP-GW, we cloned the presumptive promoters of several genes with previously described expression patterns (Table 1) into these vectors to create a set of Gateway-compatible tissue-specific plant expression vectors. We chose this set of representative promoters to allow for diverse expression patterns, including ubiquitous expression throughout the plant body, generalized root and shoot expression, cell-type-specific expression in root and shoots, and expression in specific root tissues.

**Table 1:** **Presumptive promoter regions used in vector set**

**Name**	**Gene**	**Region used in construct**	**Expected tissue expression**	**References**
*ADH1*	At1g77120	−1092 to -1	root, anoxic tissues	[25,26]
*CAB1*	At1g29930	−2148 to -1	shoot, photosynthetic tissues	[15,16]
*COBL1*	At3g02210	−730 to -5	lateral root primordia, columella, leaf vascular tissue and hydathodes	[33]
*EXP7*	At1g12360	−1866 to -1	root trichoblast	[34]
*LBD16*	At2g42430	−1309 to +1	lateral root primordia	[35-37]
*SCR*	At3g54220	−2162 to +1	root endodermis, endodermis initials, quiescent center, shoot apical meristem L1 layer, shoot endodermis, shoot tissue surrounding vascular bundles	[30-32]
*UBQ10*	At4g05320	−1612 to -28	throughout the plant	[14]
*WOL*	At2g01830	−2085 to +1	root vascular cylinder and pericycle	[38,39]

#### Ubiquitous expression

*UBIQUITIN10* (*UBQ10*) is a transcript expressed throughout the plant and is often used as a control in expression profiling experiments. Large-scale transcript profiling experiments have revealed that *UBQ10* is stably expressed and is in the top percentile of highly expressed genes in Arabidopsis [14], suggesting that genes driven by the *UBQ10* promoter would be highly expressed throughout the plant. To create a Gateway-compatible plant expression vector for uniform expression to serve as an alternative to *35S*-driven vectors, we cloned the upstream regulatory region of *UBQ10* into pMCS:GW and pMCS:YFP-GW. These new vectors, named pUBQ10:GW and pUBQ10:YFP-GW, can be used to create constructs for expressing genes encoding untagged or YFP-tagged proteins behind the *UBQ10* presumptive promoter.

#### General shoot and root expression

We were interested in developing Gateway-compatible expression constructs for shoot and root tissues. *CHLOROPHYLL A/B BINDING PROTEIN 1* (*CAB1*) expression is light-regulated in a phytochrome-dependent manner [15,16], induced by cold treatment [17], and regulated by circadian rhythms [18,19] and sugar homeostasis [20,21]. Additionally, *CAB1* is expressed in leaf and stem tissues, but not in root tissues in peas and tobacco [22-24]. Because both near and far upstream regions of the Arabidopsis *CAB1* promoter are involved in the specificity of *CAB1* expression [22], we captured the region from -2148 to -1 (where +1 is the A of the *CAB1* ATG) upstream of *CAB1* to drive expression of our Gateway-compatible constructs. We cloned this CAB1 upstream regulatory region into pMCS:GW and pMCS:YFP-GW to create pCAB1p:GW and pCAB1p:YFP-GW.

The *ALCOHOL DEHYDROGENASE1* (*ADH1*) promoter region has previously been used to express genes exclusively in root tissues; however, *ADH1* expression in roots is not developmentally regulated. Instead, *ADH1* expression is upregulated in horizontally-grown roots on agar plates because these roots are experiencing the stress of hypoxia [25,26]. Additionally, *ADH1* expression is upregulated by dehydration [26,27], ABA treatment [27,28], cold [26,27], and is altered by space flight [29]. Because Dolferus *et al.* [26] reported the region 1 kb upstream of *ADH1* was sufficient to drive GUS reporter expression to mimic the developmental and tissue-specific expression of the endogenous *ADH1* gene, we cloned the *ADH1* upstream regulatory region from -1092 to -1 (where +1 is the A of the *ADH1* ATG) into pMCS:GW and pMCS:YFP-GW to create Gateway-compatible vectors for expression of genes in roots of horizontally-grown seedlings on agar plates. These new vectors are named pADH1:GW and pADH1:YFP-GW.

#### Tissue-specific expression throughout shoots and roots

*SCARECROW* (*SCR*) exhibits tissue-specific expression in both shoot and roots. *SCR* expression is detected in the endodermis, endodermis initials, and occasionally the quiescent center of roots [30,31]. In addition, *SCR* is expressed in the endodermis of seedling hypocotyls, in the L1 layer of the shoot apical meristem, and in the tissue layer adjacent to vascular bundles [32]. The 2.5-kb region upstream of *SCR* is sufficient to drive this expression pattern [30,32]. We cloned the *SCR* upstream regulatory region into pMCS:GW and pMCS:YFP-GW to create pSCR:GW and pSCR:YFP-GW.

*COBRA LIKE1* (*COBL1*) is expressed in several tissues throughout the root, including columella, weakly in the stele, and strongly in lateral root primordia [33]. In addition, *COBL1* is expressed in leaf vascular tissue and hydathodes [33]. The 687-bp region upstream of *COBL1* is sufficient to drive this expression pattern [33]. We cloned the *COBL1* upstream regulatory region into pMCS:GW and pMCS:YFP-GW to create pCOBL1:GW and pCOBL1:YFP-GW.

#### Tissue-specific expression in roots

Plant roots serve as an ideal developmental model because cells within an individual root are at various developmental stages, ordered from the root tip to the root-shoot junction. Root cells are also organized by radial symmetry, allowing for analysis along the radial axis. In addition to the Gateway-compatible tissue-specific vectors described above, which allows for expression in both root and shoot tissues, we created root tissue-specific Gateway-compatible vectors. We chose a representative set of promoters expressed in cell-type-specific manner within the roots, including *EXPANSIN7*, *LATERAL ORGAN BOUNDARIES-DOMAIN 16*, and *WOODEN LEG* upstream regions, and cloned them into pMCS:GW and pMCS:YFP-GW.

*EXPANSIN7* (*EXP7*) is strictly expressed in root hair cells, and is not expressed in aerial portions of the plant [34]. The 386-bp region upstream of *EXP7* was sufficient to drive this expression pattern [34]. We cloned the *EXP7* upstream regulatory region into pMCS:GW and pMCS:YFP-GW to create pEXP7:GW and pEXP7:YFP-GW.

The enhancer trap line J0192 [35] has been used to drive expression of genes in lateral root primordia [36]. The insertion in J0192 is upstream of *LATERAL ORGAN BOUNDARIES-DOMAIN 16* (*LBD16*) [35]. *LBD16* is predominantly expressed in roots [37]. In young seedlings, *LBD16* is expressed strongly in the primary root tip and at the root-shoot junction [35], whereas, in older seedlings, *LBD16* expression is restricted to young lateral root primordia (Stages I-IV) and is no longer detected in root tips [35]. Additionally, *LBD16* is weakly expressed in the root vasculature [35]. The 1.5-kb region upstream of *LBD16* is sufficient to drive this expression pattern [35]. We cloned the *LBD16* upstream regulatory region into pMCS:GW and pMCS:YFP-GW to create pLBD16:GW and pLBD16:YFP-GW.

*WOODEN LEG*/*CYTOKININ RESPONSE1*/*ARABIDOPSIS HISTIDINE KINASE4* (*WOL*/*CRE1*/*AHK4*) is expressed in the vascular cylinder and pericycle of root tissues [38,39]. We cloned the *WOL* upstream regulatory region into pMCS:GW and pMCS:YFP-GW to create pWOL:GW and pWOL:YFP-GW.

These tissue-speficic-promoter-driven Gateway-compatible constructs allow for easy insertion of genes of interest and can be used to drive expression of genes of interest in various root and shoot tissues.

### Evaluation of tissue-specific expression

**Figure 2:**
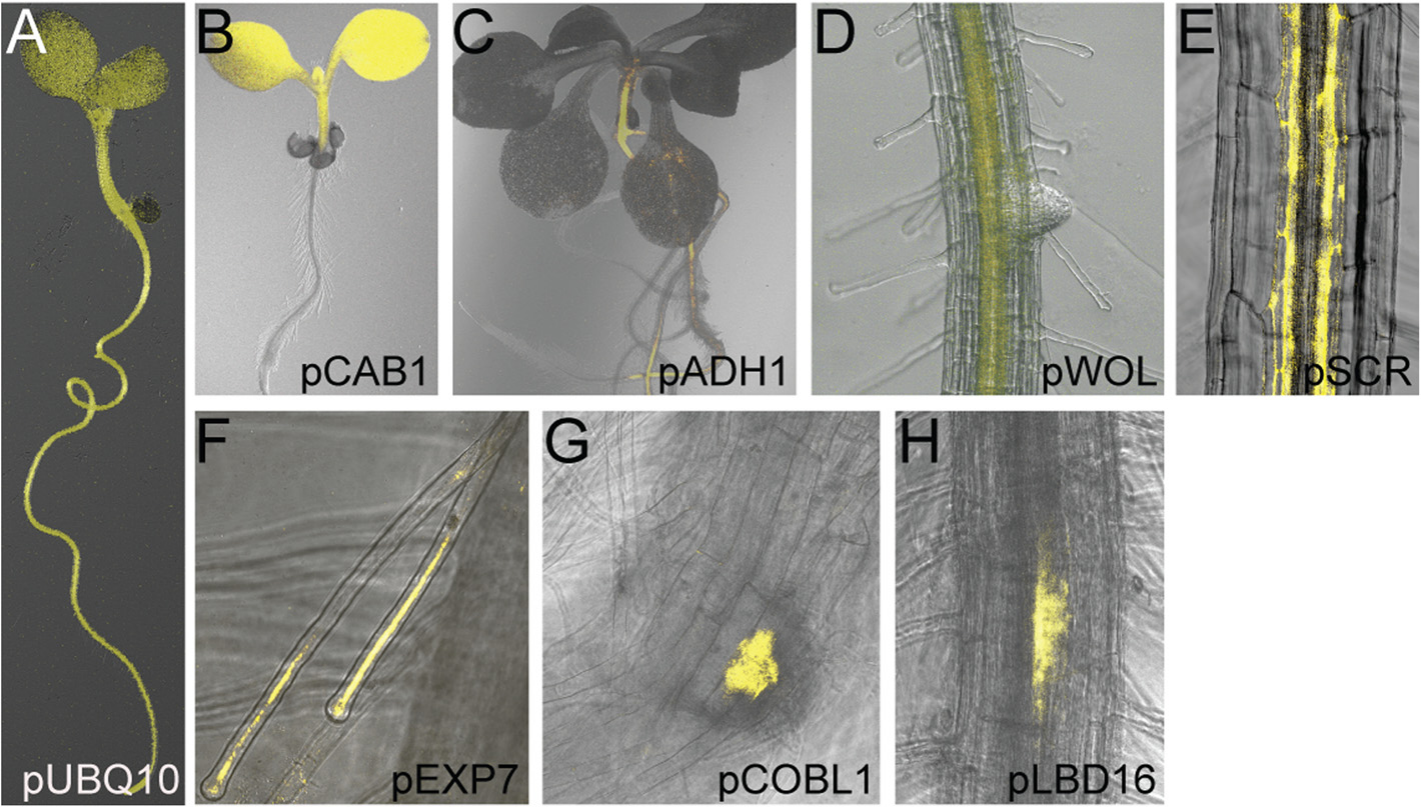
**Tissue-specificity of pUBQ10:YFP-GW, pCAB1:YFP-GW, pADH1:YFP-GW, pWOL:YFP-GW, pSCR:YFP-GW, pEXP7:YFP-GW, pCOBL1:YFP-GW, and pLBD16:YFP-GW. (A)** YFP signal is detected in all examined tissues of seedlings carrying the pUBQ10:YFP-GW vector by epi-fluorescence microscopy. Epi-fluorescence microscopy reveals shoot and root YFP signal in seedlings carrying **(B)** pCAB1:YFP-GW and **(C)** pADH1:YFP-GW, respectively. **(D)** YFP signal is detected in the vascular bundle and pericycle of seedlings carrying the pWOL:YFP-GW vector by epi-fluorescence microscopy. Confocal microscopy reveals YFP signal in **(E)** root endodermis of seedlings carrying the pSCR:YFP-GW vector, **(F)** root hairs of seedlings carrying pExp7:YFP-GW, **(G)** lateral root primordia of seedlings carrying pCOBL1:YFP-GW, and **(H)** lateral root primordia of seedlings carrying pLBD16:YFP-GW.

To determine whether our Gateway-compatible tissue-specific expression constructs expressed in the predicted (see Table 1) tissues, we transformed each of them into the Col-0 background. We found that the Gateway cassette, present in each of these constructs and carrying the ccdB “death” gene, had no obvious effects on plant growth and development. pUBQ10:YFP-GW expression was detected throughout the plant (Figure 2A) whereas pCAB1:YFP-GW expression was detected only in the shoot (Figure 2B). We found that, as expected [25,26], we could detect YFP signal only in roots of horizontally-grown seedlings expressing pADH1:YFP-GW (Figure 2C). We further detected signal from pWOL:YFP-GW in the vascular bundle and pericycle (Figure 2D). We detected signal from pEXP7:YFP-GW in root trichoblast cells (Figure. 2E). The YFP signal was detected in lateral root primordia of seedlings transformed with the pCOBL1:YFP-GW (Figure 2F) and pLBD16:YFP-GW (Figure 2G) constructs. Seedlings transformed with pSCR:YFP-GW displayed fluorescence in the root endodermis (Figure 2H).

Our observed expression patterns from all tested constructs matched expected expression patterns (Table 1), suggesting that these Gateway-compatible vectors will allow spatial examination of the effects of genes of interest.

## Conclusion

In summary, we have generated two plant expression vectors with multiple cloning sites upstream of a Gateway cassette for expression of either untagged or YFP-tagged genes of interest. We have further developed an initial set of ten Gateway-compatible tissue-specific gene expression vectors that allow for the expression of YFP-tagged or untagged proteins driven by the *ALCOHOL DEHYDROGENASE1*, *CHLOROPHYLL A/B BINDING PROTEIN 1*, *COBRA LIKE1*, *EXPANSIN7*, *LATERAL ORGAN BOUNDARIES-DOMAIN 16*, *SCARECROW*, *UBIQUITIN10*, and *WOODEN LEG* presumptive promoters. These vectors provide an invaluable resource to the plant community to allow for rapid generation of tissue-specific plant expression constructs.

Although our observed expression patterns (Figure 2) matched our expected expression patterns (Table 1), the tissues in which these constructs are expressed may be affected by growth conditions and mutant background. For example, the *ADH1* promoter, which responds to anoxia for root expression [25,26] is also responsive to dehydration [26,27], ABA treatment [27,28], cold [26,27], and space flight [29]. Thus, caution and appropriate controls should be used when interpreting data.

We anticipate that additional tissue-specific Gateway-compatible vectors based on the MCS:GW and MCS:YFP-GW vectors will be created by our lab and by other labs. We will maintain a database of further constructs created from the MCS:GW and MCS:YFP-GW backbones at http://pages.wustl.edu/strader/vectors.

In summary, we have engineered a set of Gateway-compatible vectors for tissue-specific expression to provide a reliable cloning method for quick creation of expression constructs for multiple tissues. We anticipate that these vectors will be a useful addition to the many Gateway-compatible vectors currently available to the plant community.

## Materials and methods

### Vector construction

#### pMCS:GW and pMCS:YFP-GW vectors

To remove the *Eco*RI site from the chloramphenicol resistance gene region of pEarleyGate100 [12] and pEarlyGate104 [12], we performed site-directed mutagenesis using the QuikChange Lightning Multi Site-Directed Mutagenesis Kit (Agilent) and primers CM-M1 (5′ – CATCCGGAGTTCCGTATGGCAATGAAAGACGGTGAGCTG – 3′) and CM-M2 (5′ – CAGCTCACCGTCTTTCATTGCCATACGGAACTCCGGATG – 3′)*.* To remove the *Xho*I and *Eco*RI sites from the YFP region of pEarlyGate104 [12], we performed site-directed mutagenesis using the QuikChange Lightning Multi Site-Directed Mutagenesis Kit (Agilent) and primers YFP-M1 (5′-GACTCAGATCACGAGCTCAAGCTTCAAATTCTGCAGTCGACGGTA-3′) and YFP-M2 (5′-TACCGTCGACTGCAGAATTTGAAGCTTGAGCTCGTGATCTGAGTC-3′). To create a double-stranded multiple cloning site (MCS), we annealed MCS-1 (5′-AATTCGCGACGTCCCTAGGAGATCTTCGAACACGTGAGGCC-3′) and MCS-2 (5′-TCGAGGCCTCACGTGTTCGAAGATCTCCTAGGGACGTCGCG-3′) by heating 100 pmol of each oligonucleotide in 2x SSC to 100°C for 5 minutes before cooling to room temperature on the bench top. This annealed double-stranded MCS had overhangs compatible with *Eco*RI and *Xho*I restriction sites. The mutant pEarleyGate100 and pEarlyGate104 vectors were digested with *Eco*RI and *Xho*I to excise the *35S* promoter. The double-stranded MCS was cloned into the mutant pEarleyGate100 and pEarlyGate104 backbones to create pMCS:GW (derived from pEarleyGate100) and pMCS:YFP-GW (derived from pEarleyGate104).

#### Cloning upstream regulatory regions

Genomic DNA was extracted from *Arabidopsis thaliana* Columbia-0 (Col-0) seedling tissue [40]. Upstream regulatory regions for use in tissue-specific expression vectors were PCR-amplified from Col-0 genomic DNA using *Pfx* Platinum Taq (Life Technologies) and the following primer pairs: ADH1-*Eco*RI (5′-GAATTCCACACTGAAGAAAAAGATTACACC-3′) and ADH1-*Xho*I (5′-CTCGAGCAACAGTGAAGAACTTGCTTTTG-3′); CAB1-*Nru*I (5′-TCGCGAGACTAACTTGTGAGTGAGAGTG-3′) and CAB1-*Xho*I (5′-CTCGAGGAGGTTGAGTAGTGCAGCAC-3′); COBL1-*Nru*I (5′-TCGCGACTCATGTTTGGTTGTACTACTG-3′) and COBL1-*Xho*I (5′-CTCGAGCTGAAGCAAAAAAAGAGAGAG-3′); EXP7-*Eco*RI (5′-GAATTCCGTCAAGGCTGGATATGCTGTG-3′) and EXP7-*Xho*I (5′-CTCGAGGCTGCGATCTAACAATTTCAGAC-3′); LBD1-*Eco*RI (5′-GAATTCGCGGAAGAACTTATAAAATAAC-3′) and LBD1-*Xho*I (5′-CTCGAGCGGCGAAACGAACAAAAAAGTG-3′); SCR-*Eco*RI (5′-GAATTCGATTGTGATCCTCTGCAACAAAGC-3′) and SCR-*Xho*I (5′-CTCGAGGGAGATTGAAGGGTTGTTGGTCG-3′); UBQ10-*Aat*II (5′-GACGTCGTATGATCGCGAAGCACCCACCCTAAGC-3′) and UBQ10-*Xho*I (5′-CTCGAGGACAAATTCGATCGCACAAAC-3′); and WOL1-*Aat*II (5′-GACGTCCTCACACACCACACCATCATTATC-3′) and WOL1-*Xho*I (5′-CTCGAGCACTTCAAATGTAGGTATTCC-3′). The resulting PCR products were captured into the pCR4 vector (Life Technologies) to create pCR4-ADH1p, pCR4-CAB1p, pCR4-COBL1p, pCR4-EXP7p, pCR4-LBD16p, pCR4-SCRp, pCR4-UBQ10p, and pCR4-WOL1p. All constructs were sequenced (GeneWiz, Inc.) to confirm error-free clones.

#### Gateway-compatible tissue-specific expression vectors

The *ADH1* upstream regulatory region was excised from pCR4-ADH1p using *Eco*RI and *Xho*I and subcloned into pMCS:GW and pMCS:YFP-GW to create pADH1:GW and pADH1:YFP-GW. The *CAB1* upstream regulatory region was excised from pCR4-CAB1p using *Nru*I and *Xho*I and subcloned into pMCS:GW and pMCS:YFP-GW to create pCAB1:GW and pCAB1:YFP-GW. The *COBL1* upstream regulatory region was excised from pCR4-COBL1p using *Nru*I and *Xho*I and subcloned into pMCS:GW and pMCS:YFP-GW to create pCOBL1:GW and pCOBL1:YFP-GW. The *EXP7* upstream regulatory region was excised from pCR4-EXP7p using *Eco*RI and *Xho*I and subcloned into pMCS:GW and pMCS:YFP-GW to create pEXP7:GW and pEXP7:YFP-GW. The *LBD16* upstream regulatory region was excised from pCR4-LBD16p using *Eco*RI and *Xho*I and subcloned into pMCS:GW and pMCS:YFP-GW to create pLBD16:GW and pLBD16:YFP-GW. The *SCR* upstream regulatory region was excised from pCR4-SCRp using *Eco*RI and *Xho*I and subcloned into pMCS:GW and pMCS:YFP-GW to create pSCR:GW and pSCR:YFP-GW. The *UBQ10* upstream regulatory region was excised from pCR4-UBQ10p using *Aat*II and *Xho*I and subcloned into pMCS:GW and pMCS:YFP-GW to create pUBQ10:GW and pUBQ10:YFP-GW. The *WOL1* upstream regulatory region was excised from pCR4-WOL1p using *Aat*II and *Xho*I and subcloned into pMCS:GW and pMCS:YFP-GW to create pWOL1:GW and pWOL1:YFP-GW. All constructs were sequenced (GeneWiz, Inc.) to confirm error-free clones.

### Plant transformation and plant growth conditions

The pADH1:YFP-GW, pCAB1:YFP-GW, pCOBL1:YFP-GW, pEXP7:YFP-GW, pLBD16:YFP-GW, pSCR:YFP-GW, pUBQ10:YFP-GW, and pWOL:YFP-GW plasmids were electroporated into *Agrobacterium tumefaciens* strain GV3101 [41]. Arabidopsis thaliana Col-0 was transformed by the floral dip method [42]. Transformants were selected on plant nutrient (PN) medium [43] solidified with 0.6% (w/v) agar and supplemented with 7.5 μg/mL Basta (phosphinothricin).

### Microscopy

Seedlings were mounted and imaged using either a Leica MZ10F fluorescence stereomicroscope with a YFP filter set (510/20 nm excitation, 560/40 nm emission) or with a Zeiss LSM510 laser scanning microscope. Images were converted and merged using NIH Image software.

### Distribution of materials

All described plant expression vectors are deposited at the Arabidopsis Biological Resource Center (ABRC; www.arabidopsis.org). ABRC stock numbers, vector maps, and sequences can be found at http://pages.wustl.edu/strader/vectors.
